# AI in the Coach’s Chair: How Professional Coaches Navigate Identity and Role Ambiguity in Response to AI Adoption by Their Coaching Firm

**DOI:** 10.3390/bs16020211

**Published:** 2026-01-31

**Authors:** Gil Bozer, Silja Kotte

**Affiliations:** 1 Department of Human Resource Management, Sapir Academic College, D.N. Hof Ashkelon, Shderot 7915600, Israel; 2 Faculty of Business and Law, Aschaffenburg University of Applied Sciences, 63743 Aschaffenburg, Germany; silja.kotte@th-ab.de

**Keywords:** artificial intelligence, coaching, vocational adaptation, occupational identity, role ambiguity, automation-augmentation paradox, human-AI interaction

## Abstract

The emergence of artificial intelligence (AI) coaching challenges the professional roles and identities of human coaches, yet empirical research on this transformation remains scarce. This qualitative field study investigates how professional coaches navigate their roles following the organizational adoption of AI coaching. Drawing on the automation-augmentation paradox, occupational role identity, and role ambiguity theories, we analyzed 15 semi-structured interviews with 12 professional coaches in an Asian coaching firm, contextualized by pre- and post-interviews with the company CEO and the AI provider. Findings reveal that top-down AI implementation triggered significant role ambiguity, catalyzing both protective and expansive identity work. Coaches defended their unique human value (e.g., empathy), while simultaneously experimenting with AI, shifting their perception from threat to collaborative tool. This adaptive process enabled the emergence of distinct AI functions and new “blended” human–AI coaching models. Our resulting conceptual framework demonstrates that resolving the automation-augmentation paradox in relational professions is fundamentally an identity-driven process rather than a technical task reallocation. Furthermore, our findings demonstrate that organizationally induced role ambiguity can serve as a catalyst for professional renewal and vocational adaptation, particularly when supported by participatory leadership, thereby advancing theory and contributing new insights to the literature on technological and vocational transformation in organizational contexts.

## 1. Introduction

Global corporate investment in artificial intelligence (AI) has surged in recent years, from $14.57 billion in 2013 to $189.16 billion in 2023, with $25.2 billion going to generative AI (Maslej et al., 2024). AI refers to a collection of interrelated technologies capable of performing functions previously regarded as unique to human cognition, such as learning, interaction, and problem-solving (Walsh, 2020). While early adoption focused on automating routine tasks, AI is now reshaping knowledge-intensive professions. Its impact on professionals’ identities is particularly pronounced as it substitutes for or augments complex tasks, including decision-making processes and creative problem-solving activities (Eloundou et al., 2024; Faulconbridge et al., 2024). This technological disruption is now forcing professionals to reassess their work and renegotiate their occupational identities (Schmitt et al., 2024; Strich et al., 2021). The organizational implementation of AI thus often evokes ambivalent reactions, comprising both excitement and anxiety, that influence adaptation and openness to change (Lichtenthaler, 2020; Pei et al., 2025).

AI coaching (AIC) exemplifies this broader technological evolution as organizations seek novel means to expand the reach, accessibility, and impact of workplace coaching—an established human resource development practice (Schermuly et al., 2022). Compared to other human resource development (HRD) interventions, workplace coaching is a highly relational intervention, emphasizing a high-quality coach-coachee working alliance as a key success factor (Graßmann et al., 2019; Terblanche et al., 2024). Coaching utilizes a process-consultation approach, instead of expert-led consulting or instruction, recognizing the coachee as the expert in their context and enabling them to reach goals through reflection rather than advice (Kotte et al., 2021). Traditionally, a professional coach (internal or external, without formal supervisory authority) facilitates a collaborative, reflective, and goal-focused relationship to achieve outcomes valued by both the coachee and their organization (Bozer & Jones, 2018). In contrast, AIC employs machine-assisted, systematic processes to guide clients toward professional goals (Graßmann & Schermuly, 2021). Among current AIC technologies, chatbot coaching has gained particular prominence. In this format, conversational AI on messaging platforms emulates human dialogue, supporting coachees with both rule-based and generative components, adapting conversationally to user input (Kamphorst, 2017; Terblanche, 2020).

AIC has the potential to revolutionize the coaching market, thereby disrupting the professional identities of individual coaches. This revolution stems from AIC’s ability to scale access to coaching, improve cost-effectiveness, and deliver data-driven insights (Terblanche, 2020; Terblanche et al., 2024). However, AIC faces challenges, such as limited contextual understanding, inability to replicate human relational depth, algorithmic bias, and privacy concerns (Diller, 2024; Passmore & Tee, 2023). Critically, existing AIC research is predominantly conceptual (e.g., Graßmann & Schermuly, 2021; Terblanche, 2020, 2024), with scant empirical studies that mostly rely on hypothetical vignettes or laboratory and student samples (e.g., Diller et al., 2024; Mai et al., 2022; Terblanche et al., 2022) with limited applicability to real-life contexts (Langer & Landers, 2021). Furthermore, while a few studies have reported on coach attitudes toward AIC, often highlighting curiosity, skepticism, and identity concerns, most participants have either limited AIC experience or no direct AIC interaction, which further limits real-world transferability (e.g., Bruning & Boak, 2025; Terblanche et al., 2024). To address the gap in understanding about AIC, an urgent need exists for context-rich, empirical research that captures how professional coaches, including those in real-world organizational settings, actually experience, interpret, and adapt to it.

Our study investigates professional coaches employed by a single coaching company that offers coaching to external clients and that adopted AIC internally for all of its employees. This specific setting offers a rich context for exploring how organizational adoption of AIC catalyzes coaches’ reflection on and adaptation of their professional practices. We thereby gain a nuanced understanding of how coaches negotiate their roles and occupational identities both individually and collectively through shared sensemaking within their organization. By engaging with AIC as active users in multiple roles (e.g., as coachees using it to reflect upon their own collaboration-related issues and personal goals; as coaching professionals, using it to reflect upon their own coaching practice), they experience its impact firsthand in their work. Thus, they are not hypothetical observers, but professionals whose practice and norms are directly reshaped. As such, they engage in a dual process of protecting their unique human value from technological displacement, while also expansively adapting their roles to reinstate their value through incorporating new technological affordances, a dynamic that reflects the broader tension between technological displacement and reinstatement effects (Acemoglu & Restrepo, 2019).

To analyze how coaches adapt to AIC adoption within their organization, we draw on three complementary frameworks, each illuminating a key dimension of vocational adaptation and identity work. The *automation-augmentation paradox* (Raisch & Krakowski, 2021) captures the tension between AI automating tasks (i.e., replacing routine aspects of human work) and augmenting human capabilities, illuminating both the disruptive and enabling aspects of adopting AI in coaching. *Occupational role identity theory* (Ashforth, 2001; Stryker & Burke, 2000) provides a lens to understand how AI challenges and potentially reshapes coaches’ professional identities by examining how they construct, maintain, and adapt their self-concept as professionals (e.g., purpose, value, and expertise). Finally, *role ambiguity theory* (Kahn et al., 1964) highlights the uncertainty and psychological strains that coaches may face when their roles become fluid or contested as technology redefines their traditional boundaries and responsibilities.

At the center of our inquiry lies the overarching research question: How do human coaches negotiate their professional roles and occupational identities in relation to AIC? To answer this question, our study follows their adaptation journey: We first explore the organizational context that frames their experience, then examine the professional tensions that arise from comparing human and AI coaching, and finally investigate the emerging roles they envision for AIC.

## 2. Theoretical Background

This study draws on three complementary theoretical lenses to explore the adoption of AIC: the automation-augmentation paradox, occupational role identity, and role ambiguity. Together, these theories provide the framework guiding our analysis of coaches’ complex identity work.

### 2.1. Automation-Augmentation Paradox

The automation-augmentation paradox serves as a central lens for understanding the adoption and experience of AI in knowledge-intensive, relational professions such as coaching, especially within an organizational context. This framework distinguishes between two fundamental approaches to AI deployment: automation and augmentation (e.g., Autor, 2015; Jarrahi, 2018; Raisch & Krakowski, 2021). Automation involves using AI systems to perform rote tasks with maximal efficiency and standardization, while augmentation emphasizes the complementary, technology-enabled enhancement of human capabilities, workflows, and innovation (Wilson & Daugherty, 2018).

Early literature often framed automation and augmentation as mutually exclusive and conflicting strategies, frequently depicting augmentation as inherently superior. However, recent research reveals they have a more nuanced and interdependent relationship (Raisch & Krakowski, 2021). Automation and augmentation in fact frequently coexist and interact, generating paradoxical tensions within jobs. Furthermore, they often operate at the level of individual tasks within jobs, rather than throughout entire occupations; thus, automating some tasks can benefit human contribution in others. For instance, automating passenger demand algorithms for Tokyo taxi drivers freed them from downtime waiting for fares, effectively enhancing their productivity in the primary task of driving (Agrawal et al., 2023). Similarly, AI in radiology triages basic image screening, enabling clinicians to prioritize expert diagnosis and patient care and thereby augmenting their expertise (Najjar, 2023). Initial findings on AIC indicate that AI chatbot coach assistants can help track goal progress through regular check-ins and reminders, enabling human coaches to ‘go deeper’ with higher-order complex coaching activities (Terblanche et al., 2024).

From a paradox theory perspective, automation and augmentation represent interdependent logics, whose value and impact are context dependent, including the evolving nature of work (Schad et al., 2016). The cyclical interplay between automation and augmentation (Raisch & Krakowski, 2021) holds particular relevance in the context of AIC adoption. For example, automating specific coaching tasks, such as goal setting and progress monitoring, can augment human coaching by providing personalized suggestions for future sessions. Conversely, such augmentation may yield interaction data that can be used to train algorithms to automate exercise recommendations for clients with similar profiles. Thus, data outputs from augmentation become inputs for automation, creating a self-reinforcing cycle in which each approach continuously bolsters the other (Beane & Orlikowski, 2015). Thus, being confronted with AIC, coaches live this paradox daily as they must continually discern which elements of their practice are supplanted or reconfigured and which are enhanced or made uniquely valuable.

The automation-augmentation framework is particularly useful for understanding how organizationally embedded coaches experience the paradoxical nature of AIC, viewing it as both a potential threat to their core professional identity and a valuable tool for practice enrichment and career development. This lens enables exploring how coaches and their organizations define AIC’s roles, navigate their own evolving function, and manage the inherent tension between technological gains and human-centered value. Ultimately, our study illuminates how professional coaches reconcile the pressures produced by technological innovation in AI with the core attributes of their vocational identity.

### 2.2. Occupational Role Identity

Occupational role identity theory explains how professionals develop and maintain a self-concept rooted in and shaped by the norms, values, and boundaries of their vocation (Ashforth, 2001; Stryker & Burke, 2000). This identity shapes not only how individuals see themselves, but also how they interpret professional challenges and changes (Ibarra, 1999; Pratt et al., 2006). Traditionally, role identity is reinforced through stable routines, expertise, and established relational norms within the profession. However, role identity is dynamically reshaped by environmental upheavals, such as AI adoption, that can challenge those foundational elements (Selenko et al., 2022; Sergeeva, 2023).

AI technologies, especially in knowledge-intensive fields such as coaching, are transforming core work practices by actively shaping and sometimes redefining the boundaries of expert judgment, client relationships, and professional norms (Faulconbridge et al., 2024; Strich et al., 2021). As AI-driven processes change which tasks are performed, how they are delivered, and what constitutes professional value, coaches may experience ‘work-identity integrity violations’ whereby their self-concept as professionals comes into conflict with the roles and expectations imposed by technological adoption—that is, mismatches between ‘who I am’ and ‘what I do’ (Petriglieri, 2011). For instance, having to engage with AIC can push coaches toward feeling like ‘technology administrators’, potentially creating distance from the relational, human-centered practice at the heart of their vocational identity (Terblanche et al., 2024). AI can enhance expertise, autonomy, and service quality, but it also poses risks, including deskilling, loss of meaning, decreased empathy, and challenges to professional legitimacy, depending on how it is implemented and on the context of its adoption (Sartirana & Domenico, 2025). The resulting psychological tensions reflect the struggle to maintain unique human contributions amidst shifting organizational expectations and technology-driven change (Selenko et al., 2022).

To resolve such tensions, professionals often engage in ‘identity customization,’ a process of consciously restructuring their vocational boundaries and self-concept. This customization uses three primary strategies (Pratt et al., 2006): (a) *enriching* identity by adding new skills or responsibilities (e.g., a coach learning data analytics to improve their coaching by data-driven insights on conversational parameters such as conversation shares, i.e., becoming a ‘data-informed coach’); (b) *patching* by blending overlapping roles (e.g., a coach with an IT background merging their two identities by advising a company on the design of an AIC tool); or (c) *splinting* by temporarily retreating to a former familiar identity to cope with current ambiguity (e.g., a coach with a background in psychodynamic therapy placing emphasis on working with the unconscious as a uniquely human ability and thereby discrediting AIC). While these mechanisms are well documented in highly regulated and specialized fields such as radiology (Perez et al., 2024) and banking (Strich et al., 2021), coaching presents distinct challenges. It has diverse certification standards, less formalized professional boundaries, and varied practitioner backgrounds (Sime & Jacob, 2018), and adaptation to AIC may require different or novel adaptive strategies.

The emergence of AIC compels coaches to renegotiate the core of their professional value, traditionally grounded in reflective dialogue, authentic human connection, and empathy (Nobes, 2025). While this foundational ‘humanness’ is often positioned as an irreplaceable aspect of coaching, empirical debate questions its perceived superiority. Recent meta-analytic evidence from studies in which the message source is concealed suggests AI-generated messages can be rated as more empathic than human ones (Ong et al., 2025). Navigating this complex tension, coaches may protect enduring human-centric skills while embracing new competencies related to technology use or data-informed practices.

Ultimately, adapting occupational identity in the face of AI is not a single event, but a dynamic process of negotiation. It most frequently begins with navigating the initial defensive reactions to identity threats and uncertainty and evolves toward proactive boundary setting, narrative creation, and ongoing sensemaking (Selenko et al., 2022; Sergeeva, 2023). In coaching, these processes are driven by both individual reflection and collective discourse, occurring within the coaches’ organization and extending to shared conversations among stakeholders within the broader coaching industry. Applying the lens of occupational role identity thus focuses attention on how coaches interpret and respond to AI-driven changes—not only defending tradition but also reconstructing professional relevance and expertise in a rapidly evolving landscape.

### 2.3. Role Ambiguity

As professional and occupational boundaries are reshaped by technology, professionals confront role ambiguity when they lack clarity about their job boundaries, responsibilities, or performance expectations; this ambiguity can be exacerbated by technological disruptions (Kahn et al., 1964; Rizzo et al., 1970). In the present study’s setting, the coaching company’s decision to adopt AIC compelled all its coaches to directly interact with the technology, experiencing AIC firsthand as active users, while simultaneously maintaining their role as coaching professionals, providing human coaching themselves. This simultaneous exposure was a specific driver of role ambiguity as it introduced new dimensions of uncertainty, not merely about job descriptions, but about the evolving boundaries between human and machine roles, the locus of expertise and legitimacy, and the allocation of decision-making authority (Jarrahi, 2018; Strich et al., 2021).

For these full-time organizationally employed coaches, such ambiguity could manifest as emotional strain, anxiety, or questions of self-worth and professional identity, particularly as the distinctiveness of their contributions, legitimacy in the eyes of clients and colleagues, and future role security feel newly uncertain (Diller et al., 2024; Tang et al., 2022). Furthermore, this uncertainty may be heightened by the collective nature of the AIC rollout, where, instead of incremental, individualized adoption, ambiguity unfolds in shared, company-wide dialogue and adaptation.

Although research has primarily linked role ambiguity to negative outcomes, such as increased stress, diminished job satisfaction, and reduced performance (e.g., Kern & Zapf, 2021; Nazareno & Schiff, 2021), in the presence of participatory leadership and collective recognition, ambiguity can foster learning, professional reinvention, and adaptive identity work during organizational change (Martínez-Díaz et al., 2020). In the context of AIC, role ambiguity is particularly pronounced as coaches and their organization collaboratively renegotiate boundaries between human and machine coaching, determine appropriate task allocation, and reassess client and management expectations. Accordingly, clarifying task boundaries, reinforcing core professional competencies, and applying blended implementation models (e.g., combining human and AI coaching) can help address these challenges (Faulconbridge et al., 2024; Lebovitz et al., 2022).

By analyzing how coaches perceive and navigate role ambiguity arising from their ongoing uncertainty about boundaries, distinctive value, and the evolving needs of clients, this study offers a critical lens for understanding professional adaptation and expertise negotiation as AI reshapes workplace coaching.

### 2.4. Synthesis and Research Questions

**Figure 1:**
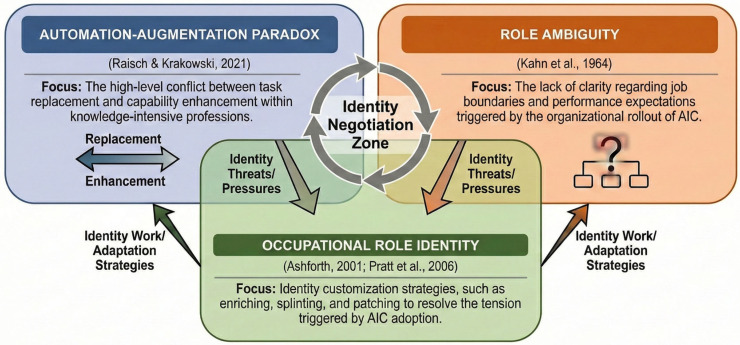
Integration of the three theoretical frameworks (Raisch & Krakowski, 2021; Kahn et al., 1964; Ashforth, 2001; Pratt et al., 2006).

Taken together, these three theoretical frameworks guide our investigation into how coaches’ organizational embeddedness, as well as their dual perspective as active users of AIC and coaching professionals, shape the tensions, boundary negotiations, and redefinitions of professional identity that emerge in their everyday practice. While our primary lenses focus on the psychological and relational impacts of AIC engagement, we situate these experiences within the broader organizational context using the Consolidated Framework for Implementation Research (CFIR; Damschroder et al., 2009). CFIR allows for systematic categorization of the organizational goals, a key intervention characteristic, the procedural rollout, an essential process component, and employee responses to AIC adoption, essential individual and collective stakeholder characteristics. Figure 1 provides a synthesis of our three theoretical lenses.

Through interviews with professional coaches employed by an Asian coaching company that has adopted AIC, we explore three interconnected questions. Through our first research question (RQ1), we set the stage for analyzing the specific organizational environment in which identity work takes place and the sources of role ambiguity: How does the organizational context of AIC adoption shape coaches’ experience of AIC? We then probe the central tensions at the heart of their practice with our second research question (RQ2): How do human coaches compare their own coaching practices to AIC? This question addresses the automation-augmentation paradox and explores the core challenges to coaches’ occupational role identity. Finally, we explore the outcomes of coaches’ identity negotiation through our third research question (RQ3): What roles and functions do human coaches ascribe to AIC? This question examines how coaches engage in boundary work to resolve ambiguity, possibly leading to the emergence of new, blended (AI-human) professional models of coaching delivery.

## 3. Methods

### 3.1. Research Design

We used a qualitative research design to explore how professional coaches experience and navigate role adaptation and occupational identity negotiation as their organization adopts AIC. Qualitative methodology is a strong choice for exploring and capturing the rich, nuanced accounts of individual-level experiences and meaning-making, which are critical for understanding emergent processes such as identity work, adaptation, and boundary negotiation in novel contexts like the adoption of AI technologies (Edmondson & McManus, 2007; Gioia et al., 2013). Unlike quantitative designs or hypothetical scenarios, qualitative inquiry allows for in-depth investigation of how adaptation to AIC occurs in real organizational settings. In addition, it can incorporate the unique contextual factors that shape human coaches’ experience of AIC adoption. To fully understand how coaches interpret, negotiate, and adapt their roles and professional identities as AIC becomes integrated into their daily work, it is crucial to capture both the organizational context of their coaching company, which is itself situated in a transforming coaching market, and the fundamental technological transformation, in particular, the accelerating AI developments that the professionals face.

This study received ethical approval from the institutional review board of the second author.

### 3.2. Research Setting

The study site was an Asian coaching company that had recently implemented AIC for its 130 certified coaches. The company, specializing in executive coaching, development of internal coaches, and coach-facilitated workshops, primarily serves large corporations. The company’s established track record in coaching innovation and its proactive adoption of AIC provided an especially compelling context for an in-depth exploration of how real-world coaches, with early firsthand experiences of AIC, perceive and negotiate their roles and identities in relation to AIC within an organizational setting. At the time of the study, the company had implemented AIC in the form of chatbot coaching internally for all its employed coaches as a preparatory phase prior to offering the service to its external client organizations.

#### 3.2.1. Technical Architecture and Interaction Design

The technical architecture of the AIC platform was developed by a Scandinavian startup (the AIC provider) that provides chatbot coaching to organizational clients (B2B). It utilizes a hybrid approach that combines rule-based and generative AI. The rule-based algorithmic elements govern the overall flow of the coaching process and the deployment of a specialized coaching exercises library, while the generative AI (GPT-4o) facilitates personalized conversational guidance, feedback, and adaptive flow. Coaching is text-based and accessible to users via common enterprise platforms such as Microsoft Teams and Slack, or through a proprietary web application. The coaching process follows a structured six-stage framework (Grant & O’Connor, 2022) encompassing: (1) issue identification, (2) developmental goal setting, (3) action planning, (4) implementation support, (5) monitoring and evaluation, and (6) assessment of goal attainment. During individual 30-min sessions, the chatbot checks on the progress made since the previous interaction before guiding the coachee through these stages.

#### 3.2.2. Theoretical Foundations and Coaching Approach

The AIC’s intervention logic is grounded in a synthesis of evidence-based theoretical concepts and methodologies designed to replicate the multi-dimensional nature of human coaching. For example, to establish an emotionally safe space that fosters coachee receptivity to change, the system integrates empathy frameworks and client-centered principles (e.g., Rogers, 1951), while utilizing double-loop learning principles (e.g., Argyris & Schön, 1974) to encourage critical reflection. For goal setting and action planning, the platform utilizes the GROW model (Whitmore, 1992), Cognitive Behavioral Therapy (e.g., Beck, 1976), and solution-focused approaches (De Shazer, 1988). To incorporate context, it draws on systemic concepts and includes systemic questions to address relational dynamics, ensuring that individual development is embedded within the broader organizational system (Senge, 1990).

To fully contextualize the study, we engaged in several preparatory and background meetings at the onset of the research collaboration. Meetings with the AIC provider, including sessions with founders and the development team, provided an in-depth understanding of the AI system and its customization for the coaching company. We also accessed the AIC provider’s platform for trial sessions to experience its functionality firsthand. Further insights were gained from attending a preparatory meeting and presentation by the coaching company, as the context shapes individual coaches’ experience of AIC adoption.

### 3.3. Participants and Procedure

We adopted a purposeful and criterion-based sampling strategy (Creswell & Poth, 2018) to recruit participants with direct experience of using the AIC adopted by their company. Inclusion criteria required that participants (1) were employed as certified coaches within the organization, and (2) had completed at least one AIC session as a user. The final sample included 12 coaches (four male, eight female; ages 23–66, *M* = 42; see Table 1 for demographics, organizational roles, tenure, coaching experience, and AIC engagement), all of whom were professional coaches with higher education qualifications, with both organizational tenure and coaching experience ranging from 1 to 26 years (*M* = 9.2/8.8 years). For confidentiality, all participants were allocated a pseudonymized ID throughout the study.

To provide broader contextual insight and inform our analysis of coaches’ identity work, the sample also included the CEO of the coaching company, a certified executive coach, and the project manager from the AIC provider. Both were interviewed twice (pre- and post-AIC adoption), enriching our understanding of the organizational context (e.g., motivations and strategic objectives for AIC adoption).

Participants were recruited through an introduction by the AIC provider’s CEO, who acted as the project manager and primary liaison. To ensure diverse perspectives, recruitment targeted both enthusiastic AIC users and critical adopters who had discontinued use early but had participated in at least one 30-min AIC session.

### 3.4. Data Collection

We presented the study’s objectives, ethical safeguards (including ethical approval, data security, and participant anonymity), and the research procedures to both the AIC provider’s CEO and the coaching company’s R&D leader. We sent personalized email invitations directly to coaches in the company, detailing the study’s aim, confidentiality provisions, voluntary participation, data protection procedures, and our independent status (i.e., no commercial relationship with the coaching company or the AIC provider). All participants signed consent forms prior to the interviews, and data security was assured through anonymization of transcripts and secure storage of audio-recordings.

We conducted 15 semi-structured remote interviews in English between September 2023 and September 2024 via videoconferencing. Interviews lasted 29–53 min (*M* = 42 min) and were audio-recorded and transcribed verbatim with participant consent (Dresing & Pehl, 2020), yielding 171 transcript pages. Sample size was not predetermined by fixed quotas, but rather by the principles of information power and saturation (Hennink et al., 2017; Malterud et al., 2016). Our sample possesses high information power owing to the specificity of the study’s focus and its participants, namely, certified coaches with direct experience of AIC adoption in a single organization. To ensure a rigorous standard and a deep, nuanced understanding of themes, we conducted interviews until reaching meaning saturation, defined as the point at which no new themes or analytic categories emerge (Anderson, 2017; Saunders et al., 2018). Saturation was reached after the 11th coach interview. Triangulating coach interviews with the perspectives of the coaching company’s CEO and the AIC provider further enhanced the analytic rigor and contextual relevance of the findings.

Interview questions were developed to align with the research questions and were informed by workplace coaching frameworks (e.g., Ely et al., 2010; Greif, 2013; Kotte & Bozer, 2022), AIC literature (e.g., Graßmann & Schermuly, 2021; International Coaching Federation, 2024; Terblanche, 2020), and three key frameworks (the automation-augmentation paradox, e.g., Raisch & Krakowski, 2021; occupational role identity, e.g., Ashforth, 2001; Pratt et al., 2006; and role ambiguity, e.g., Kahn et al., 1964; Rizzo et al., 1970).

We developed separate semi-structured interview guides for the three stakeholder groups: coaches, the CEO of the coaching company, and the AIC provider’s project manager. Each guide combined predetermined questions with open-ended, narrative prompts to ensure consistency, while allowing in-depth exploration of emergent topics. Storytelling techniques were integrated to facilitate richer, nuanced accounts, encouraging participants to recall and describe specific moments from their experiences with the AIC, such as their initial introduction to AIC, or to reflect on particularly positive or negative incidents during their interaction with AIC (Lewis, 2011).

The coach interview guide focused on how coaches experience and respond to AIC adoption. It included questions about their professional background, engagement with AIC, memorable incidents (e.g., “Can you describe a specific moment or incident during your AI coaching sessions that felt particularly significant, whether positive or negative?”), and their personal goals and benefits associated with their AIC engagement. Coaches were asked to reflect on their interactions with the AI coach, compare AI and human coaching, and discuss the roles they ascribe to AIC (e.g., “As a professional human coach, how do you perceive your relationship with the AI coaching?”). Additional questions explored the organizational context, strategic motivations for AIC adoption, and the impact on the coaches’ professional roles and identities (e.g., “What were your immediate thoughts and emotions when the AIC adoption was first announced, and what specific professional concerns, if any, did you have?”). The interview concluded by inviting coaches to consider the future of AIC and its potential impact on their work and profession (e.g., “How do you anticipate the adoption of AIC will impact your coaching practice and your professional role as a coach?”).

The CEO was interviewed before and after AIC adoption to capture their evolving motivations, expectations, and reflections. The pre-adoption guide explored the CEO’s understanding of workplace coaching, reasons for AIC adoption (e.g., “What was the specific cause or impulse that motivated the decision to implement AIC at this particular time?”), organizational objectives (e.g., “What are the primary organizational objectives you hope to achieve through this project?”), and anticipated impacts. The post-adoption guide evaluated project outcomes, the extent to which objectives were met (e.g., “To what extent do you feel you have achieved your initial objectives, and how successful do you consider the implementation at this stage?”), lessons learned, and the CEO’s perspective on ongoing integration, improvements, and the future of AIC (e.g., “How do you envision the future role of AIC within your company?”).

The project manager of the AIC provider was also interviewed before and after AIC adoption to capture the service provider’s perspective on the implementation process. The pre-adoption guide addressed the initiation of the collaboration and the distinctiveness of the client organization as a human coaching service provider (e.g., “What stands out to you about this specific client organization compared to others you have worked with?”). We further explored the alignment of AIC with its business strategy and expectations (e.g., “What do you perceive as the primary trigger that prompted this organization to implement AIC for its entire workforce at this time?”). The post-adoption guide focused on reflections regarding the partnership with the coaching company, including implementation barriers and facilitators (e.g., “What factors fostered or hindered the implementation of AIC in this company?”), and outcomes (e.g., “What specific outcomes have you observed?”). We also explored the particularities of collaborating with a coaching company and human coaches as AIC recipients (e.g., “What specific factors impacted the acceptance of the AIC among these professional coaches?”). Finally, we asked about the primary topics covered in AIC and key lessons learned.

### 3.5. Data Analysis

To analyze the interviews, we conducted a Qualitative Content Analysis (QCA; Mayring, 2015; Schreier, 2012). QCA is defined as ‘category-driven, qualitatively oriented text analysis’ (Mayring, 2015, p. 30; our translation), which provides both flexibility and structure for analyzing complex, context-dependent phenomena. While QCA shares similarities with thematic analysis (TA), namely the objective of identifying recurring patterns across a dataset, we employed QCA for its more structured, category-driven approach (Schreier, 2012), relying on a highly systematic coding frame that ensures every unit of analysis is consistently categorized. In addition, QCA is particularly well-suited to the hybrid development of categories and themes (Fereday & Muir-Cochrane, 2006), both inductively from participant narratives and deductively from theoretical frameworks. This hybrid inductive-deductive approach ensures analytic rigor while allowing participants’ voices and unique sensemaking to remain central (Hsieh & Shannon, 2005; Mayring, 2015; Patton, 2002). It enabled us to deductively incorporate existing theoretical knowledge from the above-mentioned frameworks to shape our initial coding frame while developing data-driven codes and categories for largely unexplored aspects, given the nascent state of theory on individual and organizational AIC adoption.

We began data analysis by reading the interview transcripts multiple times to immerse ourselves in the data and identify initial ideas (Braun & Clarke, 2006). To ensure the credibility of our analysis, we engaged in iterative triangulation across researchers throughout the process (Lincoln & Guba, 1985; Morse et al., 2002). To create a preliminary coding frame, we first independently coded a portion of the interview transcripts and then discussed our codings and the emerging categories. Next, we continued coding separately and modified and refined the coding frame several times through ongoing discussion and communicative validation (Flick, 2010), comparing our codings, discussing differences, and refining the coding frame until reaching full agreement. For each (sub-)category, we agreed on the category name, its definition, and selected illustrative quotations. In the final step, the first author coded the remaining transcripts using the finalized coding frame, marking any unclear passages and discussing them with the second author to achieve consensus. To facilitate this coding process, we used MAXQDA 2022 (VERBI Software, 2021). This computer-assisted analysis program allowed flexible categorization and organization of our qualitative material (Eriksson & Kovalainen, 2008) and enabled a transparent, systematic tracking process and documentation that strengthened the study’s methodological rigor and trustworthiness (Sinkovics & Alfoldi, 2012).

In line with QCA’s suitability for hybrid inductive-deductive analysis, to address RQ1, we deductively developed higher-order categories informed by the Consolidated Framework for Implementation Research (CFIR; Damschroder et al., 2009). We then inductively developed codes from the data that we clustered into common categories. For RQ2, we inductively generated codes and integrated them in a second step into categories aligned with the workplace coaching literature, particularly with established components of the coaching process (e.g., working alliance, coaching techniques, time and pacing). To identify the roles and functions of AIC (RQ3), we adopted a primarily inductive, data-driven approach in line with the novelty of this topic.

In total, we identified 51 codes, clustered into 19 categories and 9 higher-order-categories. For example, for RQ1, we deductively derived three higher-order categories based on the CFIR (Damschroder et al., 2009), differentiating intervention characteristics, namely (1) strategic motivations of the organization to adopt AIC, process characteristics, namely (2) organizational processes for the introduction and communication of AIC adoption, and stakeholder characteristics, namely (3) coaches’ initial emotional and cognitive reactions. Within each of these higher-order categories, we inductively identified codes and clustered them into common categories. For RQ1, we identified 16 codes, clustered into 8 categories. For example, within the higher-order category ‘strategic motivations to adopt AIC’, we identified 8 codes. The codes ‘cost-effective scalability’, ‘new target groups’, ‘international geographical reach’, and ‘cultural acceptance’ were clustered into the category ‘expanding services and client reach’. The codes ‘user experience and familiarization’ and ‘internal validation and improvement through feedback’ were clustered into the category ‘internal testing’. The codes ‘pioneering innovation’ and ‘market differentiation and industry leadership’ were clustered into ‘positioning as an innovative industry leader’.

## 4. Findings

This section presents the core findings of our qualitative analysis, structured around the three research questions. For each question, we provide a concise direct answer to the research question, followed by a summary table of the categories and subcategories, and a detailed analytic narrative illustrated with exemplary participant quotations.

**RQ1.**  *How Does the Organizational Context of AIC Adoption Shape Coaches’ Experience?*

The organizational context of AIC adoption consists of the organization’s strategic motivations for adopting AIC (expansion of services and client reach, internal testing, and industry leadership), and the way AIC adoption is introduced and communicated to employees (a CEO-led initiative with participatory engagement and structured communication with ongoing updates). Together, these elements form the structural foundation, or “backstage”, for the broad range of coaches’ emotional and cognitive reactions to AIC adoption, ranging from enthusiasm and curiosity to skepticism and perceived threat. Table 2 provides a summary and brief descriptions of the categories and subcategories.

### 4.1. Strategic Motivations of the Organization to Adopt AIC

Three primary motivations prompted the coaching company’s decision to adopt and implement AIC: (a) expanding client reach; (b) internal testing; and (c) positioning the company as an innovative industry leader.

#### 4.1.1. Expanding Client Reach

Coaches and management consistently described AIC as a strategic vehicle for broadening the company’s service portfolio and accessing new client segments. AIC was perceived as a means to facilitate market expansion by providing cost-effective, large-scale coaching, allowing a higher number of employees to access coaching. As Coach G explained: *‘We can provide our services to more people who need coaching on a daily or weekly basis.’* Rather than focusing solely on executive levels, this expansion allows the coaching company to target new client segments underserved by human coaching, as Coach E explicitly noted: *‘So, not just the executive or manager level, but younger managers or newcomers, like new graduate employees, can also get a coach.’*

In addition, the multilingual capabilities of AIC potentially enable delivering coaching internationally, across geographical and language barriers. The coaching company CEO emphasized this potential by saying: *‘We can offer this [AIC] not just in [our primary language], but in English, French, and German… getting employees who are working outside [our country] to take AI coaching.’*

Some coaches also suggested that AIC could reduce domestic cultural barriers and stigma associated with ‘conventional’ human coaching, thus increasing coaching uptake. Coach A reflected: *‘There are still people in [our culture] who are very skeptical, suspicious about coaching, many people are allergic to counselling or one-on-one relationships. So, AI coaching might open the opportunity for coaching in the market.’*

#### 4.1.2. Internal Testing

Management implemented AIC internally to ensure rigorous internal testing before external rollout and client outreach, reflecting an iterative strategy aimed at internal acceptance and service improvement:

To ensure acceptance and proficiency, coaches first used AIC internally, familiarizing themselves with AIC, learning experientially, and preparing them for the client rollout. Coach J explained: *‘As facilitators, we need to experience AI coaching ourselves—just like we had to be coached by a human before coaching others—so we truly understand how it works for us.‘*

Internal use of AIC also enabled an ongoing feedback loop, allowing coaches to provide input for iteratively adapting, refining, and validating the AIC platform. Coach F explained: *‘We want to test this AI coaching within the company before we provide it as a paid service to our clients. So, if we all use it, then more than 150 people can give feedback to improve this service.’*

#### 4.1.3. Positioning as an Innovative Industry Leader

AIC adoption also served to demonstrate the company’s ongoing commitment to pioneering innovation and establishing market differentiation. Coaches and management framed the AIC adoption as a strategic initiative to reinforce the company’s status as an industry leader by being among the first to integrate advanced technology into its service offerings. Participants noted that this move aligns with the company’s track record as an early adopter of new services. As Coach F remarked: *‘Actually, we are very interested in new technology, so this is very new; we have no idea. We also, when we started coaching, nobody knew about coaching in [our country], so we are very keen on trying new things.’* This commitment to innovation is linked to the need for a competitive advantage and industry standing, as Coach A said: *‘We needed to differentiate ourselves and be distinctive from other companies and be the first company to provide this kind of service.’*

### 4.2. Organizational Processes for the Introduction and Communication of AIC Adoption

A combination of top-down, yet participatory leadership and structured communication activities characterized the introduction and adoption of AIC. Analysis of participant accounts revealed three key components of the rollout strategy:

#### 4.2.1. CEO-Led, Top-Down Initiative

Participants identified the CEO as the main driver of AIC adoption, with his direct involvement in communicating the project’s purpose signaling its importance. Coach F noted: *‘Our CEO took the initiative to bring this into our company and deliver the clear message that he wants everyone to really use this and for what purpose. So that was very clear and that facilitated our engagement.’*

#### 4.2.2. Participatory Leadership: Call for Engagement and Feedback

Following the introduction, employees were encouraged to interact with the AIC, share their user experience and draw on their professional expertise as coaches to provide feedback, taking an active role in AIC’s continued development. Coach H said: *‘Our CEO made it very clear, let’s try it out… give it feedback so we can develop it together.’*

#### 4.2.3. Structured and Ongoing Communication

The CEO of the coaching company and the AIC provider led an all-employee kick-off meeting to outline the project’s vision, goals, and expectations for employee participation. Communication remained constant throughout the project, with ongoing updates and feedback channels. Coach L described: *‘It was announced in the all-hands meeting, and then we also invited the CEO of the AI coaching service provider, who came to introduce. Our CEO mentioned this initiative, and he invited everybody to try out using the AI coaching.’* Coach I noted: *‘They are keeping us informed all the time on when it’s going… and we also do the announcing, PR.’*

### 4.3. Coaches’ Initial Emotional and Cognitive Reactions

When first introduced to AIC, participating coaches described complex and often ambivalent feelings and thoughts.

#### 4.3.1. Enthusiasm and Curiosity

Many coaches expressed excitement over the new technology and a strong desire to experiment with it. This enthusiasm was often linked to an open-mindedness toward technological innovation. Coach K said: *‘I like these kinds of things… when I first heard about this AI coaching, I thought, wow, this must be exciting.*’ The curiosity was shared by Coach I, who noted: *‘You know, the world of AI these days is very, very smart and it’s everywhere. So it’s about like, yeah, how is this AI helping our industry? So that’s the curiosity that I have.’*

#### 4.3.2. Strategic Professional Opportunity

Coaches’ initial reactions also included a positive cognitive appraisal of AIC as a vehicle for professional innovation and development as coaches. Some participants therefore adopted a proactive, self-directed approach, seeking to leverage AIC to enhance their own practice. As Coach J reflected: *‘I was very interested, so my mindset was how can I use my AI coach.’* This perspective was grounded in the belief that engaging with AIC is strategically necessary for sustaining long-term vocational relevance as a coach as was further articulated by Coach L, emphasizing the necessity of future-oriented adaptation: *‘I want to accumulate firsthand experience because…we cannot avoid AI coaching development… it helps me to connect with the world and explore what I can also learn from it.’*

#### 4.3.3. Skepticism and Technological Uncertainty

Skepticism and uncertainty were also prominent initial reactions for coaches, often stemming from their lack of familiarity with AI systems. Some participants expressed an inability to imagine how AIC would function in practice, which caused hesitation about its adoption. Coach C shared: *‘I couldn’t really imagine how it works, so I was a bit skeptical.’* This professional caution was echoed by Coach A, who recalled the initial atmosphere of the rollout: *‘In the beginning […] we were not sure of this technology coming into the coaching industry.’*

#### 4.3.4. Perceived Threat to Professional Identity

Initially, some coaches reacted with anxiety and fear regarding their professional identity and the possibility that AIC could devalue their work or lead to job replacement. Their apprehension stemmed from AIC’s potential to erode the core interpersonal and relational qualities central to human coaching. Coach C reflected: *‘It’s a really amazing idea, but sometimes I worry that it might take over what we do.*’ This fear of technological displacement was shared by Coach A, who admitted: *‘In the beginning, we were scared of this technology, because it makes human coaches unnecessary. If AI coaching can be super efficient and can replace human coaching, then…’*. Coach A further contextualized this threat by noting that in a *‘busy society, clients might drift toward the ease of AI and forget about the good parts about person-to-person coaching.’*

In summary, coaches’ initial reactions to AIC reflected a dynamic emotional-cognitive spectrum, ranging from enthusiasm and strategic optimism to uncertainty and vulnerability. Many participants experienced these reactions simultaneously, illustrating the tension between embracing technological innovation and safeguarding the uniquely human aspects of their coaching profession.

**RQ2.**  *How Do Human Coaches Compare Their Own Coaching to AIC?*

When comparing human coaching to AIC, participants identified core coaching techniques (e.g., questioning, summarizing) and a non-directive approach as shared features. In terms of differences, they highlighted a fundamental divergence between the relational depth characteristic of human coaching and the instrumental efficiency associated with AIC. Human coaches are regarded as indispensable for navigating emotional complexity and ambiguity, whereas AIC is considered particularly effective for immediate goal-structuring. Coaches articulated this distinction by describing human coaches as addressing “the who,” referring to holistic, person-centered depth, and AI coaching as facilitating “the how,” fostering instrumental task execution and progress. Key advantages of AIC over human coaching that coaches highlighted are the provision of a psychologically safe, neutral, and non-judgmental space and its immediate, on-demand accessibility. Table 3 summarizes these comparative dimensions.

### 4.4. Similarities Between AIC and Human Coaching

Coaches identified fundamental similarities between human coaching and AIC, highlighting that both interventions share core coaching techniques to guide a non-directive coaching process. Central to this functional similarity is the use of open-ended questioning, summarizing, and acknowledgement to foster coachee self-reflection and insight. Coach L observed that the procedural *‘coaching job’* remains consistent across both interventions, as both *‘utilize open-ended questions to help me to explore, to focus on what I want to focus on.’* This was reinforced by Coach F, who noted that both human coaching and AIC are *‘directed towards helping the client… not giving advice, not trying to teach anything, but genuinely providing questions to think together’* allowing the coachee to *‘verbalize things on their own.‘* The similarities extend to a shared approach of combining synthesis and validation. Coach C noted that the AIC’s process of summarizing and acknowledging coachee input—before posing a new question—closely resembles human coaching: *‘It would summarise… acknowledge me, and then it would give me a question.’* These shared fundamentals of non-directive inquiry and summarizing and acknowledgement establish a facilitative framework for coachee reflection in both interventions.

### 4.5. Weaknesses and Limitations of AIC

While AIC successfully replicates the procedural mechanisms of coaching, participants identified significant constraints to its effectiveness. These limitations primarily stem from AIC’s instrumental nature, creating a “depth gap” in complex coaching scenarios. Participants observed that while AIC demonstrates proficiency in facilitating target-oriented progress, it lacks the intuitive and emotional nuances required for holistic exploration and process adaptation. The following sub-sections detail the specific constraints related to conversational flexibility, relational resonance, and user-adaptive support.

#### 4.5.1. Lack of Holistic Exploration and Adaptive/Flexible Conversation Flow

Participants’ identified AIC’s highly structured, goal-focused approach as a barrier to the deep, holistic exploration that characterizes professional human coaching. With its focus on structuring thoughts, concrete goals, and task-oriented progress through systematic questioning (i.e., coaching “the how”), AIC struggled to address the complexity of the person as a whole (i.e., coaching “the who), and to navigate the ambiguity and evolving topics inherent in deeper behavioral change.

Participants highlighted that this focused, linear approach restricted the coachee to immediate topics and surface-level task organization, limiting opportunities for open exploration and creative idea development. Coach J illustrated this functional divide, stating: *‘I choose a human coach to speak freely and develop ideas […], so for an AI coach, organizing thoughts, and for the human coach, it’s developing more ideas.’* Participants also noted that AIC rarely explored the coachee’s broader life, value and aspirations. Coach L stressed AIC’s difficulty in holistically addressing a coachee’s broader personal context: *‘I think AI is very good in coaching the how […] but it is not really coaching the who, the person I am. I’ve yet to really feel AI really make me feel like I’m really being coached as a whole person.’* This lack of holistic engagement was described as a missed opportunity for fostering meaningful personal growth.

Additionally, participants described AIC’s conversational style as rigid and overly structured, feeling like a formulaic inquiry process that lacked the evolving, adaptive, and spontaneous nature of human dialogue. Coach F contrasted the spontaneous flexibility of human coaching sessions with the AI’s limited adaptability, noting that *‘As a coach, I allow my clients the freedom to verbalize their thoughts. Sometimes, they might say something and then re-verbalize something, and that ambiguity is okay in coaching. But in AI coaching, it’s pretty difficult for me as a client to go around… to say something and say, oh, sorry, that wasn’t what I wanted to say, and re-verbalize something.’* This rigidity hindered the ability to revisit topics or explore new directions, reducing responsiveness to the coachee’s evolving thoughts. This rigidity was intensified by a repetitive inquiry style. Coach D remarked that the interaction often feels overly structured and “formulaic,” explaining that *‘the communication is always a question… answer something, then comes a question. The repetition continues forever.’* Taken together, rigid structuring, narrow content focus, and insufficient conversational agility impede AIC’s ability to support person-centered discovery and holistic transformation.

#### 4.5.2. Lack of Emotional Resonance and Relational Depth

Participants identified the lack of emotional resonance and limited relational depth as fundamental limitations of AIC, particularly its difficulty in facilitating genuine connection and personalized engagement. Unlike human coaching, which employs relational attunement to foster trust and openness, AIC was perceived as lacking the attentive listening and affective capacity needed for a strong coaching alliance. This “resonance gap” suggests that while AIC could process coachee input, it struggled to act as an empathetic witness, a deficit that hinders coachee self-disclosure.

This limitation is most apparent in the AI’s limited ability to convey authentic interest. Coach K reflected on the perceived lack of empathy and emotional resonance, stating: *‘I felt that it was not interested in me […] because it didn’t show its feelings or its thoughts […]. Of course it memorized what I just said, but it didn’t show me how it empathizes or sympathizes with me.’* As AIC provided literal rather than interpretive responses, i.e., reacting to what was said but not what may have been meant, participants described the dialogue as remaining at a “surface-level,” missing the deeper, implicit meanings. Coach F noted that the AI *‘only reacts to the actual words […], We cannot go, dive deeply into the topic behind the topic,’* rather than grasping the underlying emotional nuances that human coaches naturally infer and explore.

Furthermore, AIC’s instrumental focus often prevented a deeper connection. Coach G shared that although the AI was proficient in task-related inquiry, it failed to address the broader aspects of coachees’ personality or values: *‘I feel that AI coaching is asking questions about my topic, but is not asking questions to myself or my personality. […] I feel more connected if they ask about my life, my personality.’*

Ultimately, the absence of an emotional bond from the side of the AI significantly undermines coachee motivation and accountability. Without mutual emotional attachment and the interpersonal responsibility fostered by a human relationship, participants reported greater ease in disengaging from the process. Coach H identified this as a *‘critical problem,’* noting that because there is no real *‘other’* to hold the coachee responsible, *‘it’s easy to cancel […]. so how to comply and use it consistently is a challenge.’* Consequently, the lack of relational resonance directly hinders the development of the accountability structures essential for sustained behavioral change.

#### 4.5.3. Difficulty Supporting Novice Coachees Lacking Coaching Literacy

Participants observed that AIC lacks the flexibility required to adapt its coaching approach to the needs of individuals with limited coaching literacy, i.e., novice coachees that lack the foundational understanding required for effective engagement in self-reflective processes. While human coaches can provide educational scaffolding and adapt their methods to the coachee’s experience level, participants observed that AIC operates on a structured logic that assumes users already possess the requisite skills to guide their reflective journey. They questioned AIC’s ability to guide those lacking a basic understanding of coaching. Coach D described this “leverage gap,” contrasting the human coach’s ability to prepare coachees with the AI’s high barrier to entry: *‘For human coaches, I believe the person who is not ready to take coaching session can also be a client if they have time to talk with coaches or if they are willing to learn about coaching. But for the AI coaches, if the client has no knowledge about coaching, how they can leverage that to their own goals, I think it’s very difficult to use them by themselves.’* This finding suggests that AIC lacked the adaptive flexibility to assess a coachee’s level of coaching literacy and, when necessary, teach the “rules” of reflection accordingly.

### 4.6. Strengths and Advantages of AIC

Participants also identified notable strengths of AIC compared to human coaching. These strengths were primarily related to the psychological advantages of the sense of safety and control AIC offers, as well as its operational advantages, including accessibility and scalability.

#### 4.6.1. Psychological Safety: Freedom of Expression and Neutrality

Participants identified psychological safety as a key advantage of AIC, attributing it to the absence of a human counterpart and the resulting reduction in social anxieties. This neutrality is reinforced by the coachee’s complete control over the session’s pacing, which reduces the performance pressure typically present in real-time dialogue. Coach A highlighted the resulting freedom of expression, noting: *’I don’t get nervous. I can pause as long as I want. Whereas when I’m talking with my coach […] I sometimes feel a little pressure in my mind that I need to articulate thoughts.’*

Due to the socially neutral nature of the interaction, participants felt liberated from managing status dynamics or taking into account the coach’s feelings and reactions, concerns that often necessitate “filtering” in human coaching and hinder authentic self-expression and self-focus. Coach C shared: *‘It’s so much easier to be honest with it. I don’t have to think about my relationship with the person,’* while Coach F emphasized the *‘freedom to bring whatever topic I want without the fear of how that will impact the other person.’*

The perceived neutrality also fostered a “shame-free” environment where coachees do not worry about being judged. Coach H explained: *‘I don’t need to care about how the bot perceives me, so I think it has the psychological safety.’* The sense of safety extends to the organizational context, as participants viewed AIC as a secure space for sharing sensitive information without concerns of confidentiality or adverse professional consequences. Coach G described AIC as fundamentally *’more safe’* than human coaching, particularly when discussing sensitive organizational or personal issues: *’I think it’s great we have this kind of AI coaching, because it’s built from safety. I can share freely anything I think of our company or myself. It’s not like going to happen when I have a coaching session with a human. I think there’s some border between the amount I can share with the [human] coach.’*

Consequently, the combination of user-controlled pacing, social neutrality, and the absence of interpersonal consequences supports a level of freedom of expression and self-focus that participants found difficult to achieve in socially-bound human coaching relationships.

#### 4.6.2. Accessibility and Instrumental Utility

Beyond psychological safety, participants cited AIC’s functional features and operational utility as significant advantages, particularly its on-demand availability, scalability, and resourcefulness.

Unlike human coaching, which is limited by scheduling and individual coaches’ knowledge boundaries, AIC is constantly accessible, allowing coachees to engage in coaching whenever they need it. Participants highlighted immediate availability as a primary strength of AIC, providing a level of support that human coaching cannot match. Coach C shared: *‘It was really great to have someone to just talk to, and when I wanted to talk to… for the AI coaching, I think it’s always there for me, no matter what time it is.’* By providing a low-cost, high-availability intervention, AIC’s accessibility also allows for scaling coaching across organizations to reach broader, more diverse employee populations. As Coach I stated: *‘We may have limited resources in terms of the number of coaches and budget, but if everyone can access AI, then they can have a coach. We can provide more opportunities for people to receive coaching.’*

The instrumental utility of AIC is further enhanced by AI’s ability to synthesize extensive information in real time, enhancing coaching conversations with diverse frameworks, perspectives, and resources. Coach G emphasized that while human coaches are limited by their own backgrounds, the AI’s *‘resources cannot compare to the human’s resources,’* noting: *‘Because of its resources that are connected to everywhere […], if I say something about A, the AI replies with BCDE ideas. So, I think there is more information I can get from AI coach. I can have more like broad ideas, a broad mind to explore just one thing.’* This instrumental resourcefulness allows coachees to draw from a vast knowledge base that exceeds the cognitive reach of a single human coach.

Thus, AIC enables affordable, resourceful, large-scale support and frequent interactions that are not logistically or economically feasible through human coaching.

**RQ3.**  *What Roles and Functions Do Human Coaches Ascribe to AIC?*

Direct engagement with AIC prompted coaches to fundamentally reconsider the value and integration of AI within their professional landscape. They ascribed to AIC three primary roles: delivering coaching to clients, supporting their own development as coaches, and catalyzing the evolution of the coaching market. For the delivery of coaching to clients, they demarcated the unique value of both human and AI contributions and clarified evolving role boundaries, where AIC serves to automate structured, goal-focused tasks (“the how”) whereas human expertise is reserved for complex, relational depth (“the who”). Two differing models of coaching service delivery result, a blended delivery model where a human coach and AIC collaboratively engage in joint coaching engagements with complementing roles, and a service division model where human and AI coaching serve different coaching purposes and target groups independently. Regarding their own professional journey, they ascribe to AIC the role of a development partner, providing support for continuous growth, including skill modeling, mentorship, and feedback. Regarding AIC’s role as a catalyst for the evolution of the coaching market, the majority of coaches see AIC as a door-opener that can increase the demand for ‘real’ human coaching. As summarized in Table 4, these roles and functions enabled coaches to resolve role ambiguity by proactively reconstructing their occupational identity within a transforming professional landscape.

### 4.7. AIC’s Role in Delivering Coaching to Clients

In light of the unique values coaches ascribed to human and AI coaching, they envisioned two differing models of coaching delivery to clients: a collaborative delivery model where human and AI coaches adopt complementing roles, and a service division model where humans and AI independently deliver coaching for different purposes and target groups.

#### 4.7.1. Collaborative Coaching Delivery: Blended Human–AI-Coaching

Engagement with AIC catalyzed a shift in coaches’ perceptions, moving from fearing technological displacement toward viewing the tool as a collaborative partner. Rather than threatening their roles, AIC was recognized as a resource capable of broadening professional opportunities. Coach A summarized this transition: *‘…we came to accept and we came to think that this is not something to replace human coaching, but we can collaborate with it’*.

Framing AIC as a collaborative supplement and allocating complementary tasks to human and AI coaching allowed coaches to view it as a resource that augments human capabilities without jeopardizing the uniquely human aspects of their work. This perspective helped coaches safeguard their professional identity by positioning the technology as an aid rather than a rival. Coach E echoed this sentiment: *‘I would not think AI coaching is a competitor of human coaches. I think we can use it as a supplement tool or a complement tool’.*

Participants identified integrating AI and human coaching as a strategy to deliver more impactful, continuous development solutions. They described a blended model in which human coaches facilitate sensitive, transformative changes and navigate relational complexity, while AIC is responsible for routine follow-up, progress monitoring, and providing instrumental support between sessions. This approach is considered effective for promoting sustained behavioral change through fewer, but higher-value, human interventions. Coach A illustrated the functional efficiency of this model: *‘The client takes a human coach once a month or once in two months, and in the meantime, the AI coach checks the progress. The clients can take whatever they want… express their feelings for a short time, and proceed with the next steps.’*

#### 4.7.2. Division of Coaching Delivery: Differentiating Functions, Value Propositions, and Target Groups of Human vs. AI Coaching

Participants acknowledged that human and AI coaches offer distinct value propositions, which is expected to result in a division of coaching tasks and target groups aligned with these unique contributions. As Coach H stated: *‘the value proposition that human coach has and AI coach has is so much different,’* highlighting the distinction between relational depth and instrumental utility.

Human coaches are regarded as essential for complex, sensitive, and executive-level coaching, where deep empathy, nuanced understanding, and relational sensitivity are required to navigate intricate organizational dynamics. These attributes are considered difficult for AI to replicate, particularly when addressing the coachee as a “whole person”. In contrast, AIC excels at providing scalable support for specific, structured topics and addressing well-defined challenges, focusing on the procedural aspects of goal achievement and task execution. Coach H captured this division: *‘In a workplace or in organizations that are trying to achieve more complex things… then a human coach has his functions, especially that we can be more tailored into providing a solution… a relationship to a company. But in everyday, [when we] just want to sort our thoughts, achieve personal goals, I think those kinds of things… [are] very likely to be replaced [by AIC], yeah’* Coach J added the differentiation of target groups: *‘Humans will do more of a sensitive, executive kind of coaching […] For the very sensitive, complicated executive coaching, I think a human being will be the coach for them, because these executives need really sympathy and empathy.’*

By delineating these distinct value propositions, coaches resolved role ambiguity and reframed AIC as a specialized tool for tactical support, thereby emphasizing the unique value of human relational expertise as a core component of their occupational identity.

### 4.8. AIC’s Role for Coaches’ Own Professional Development

Beyond its role in delivering coaching to clients, coaches ascribed an essential role to AIC as a catalyst for their own continuous growth and professional development. Direct engagement with the platform enabled coaches to reduce role ambiguity by conceptualizing AIC as a developmental partner that facilitates experiential learning and provides supervision-like guidance.

#### 4.8.1. Experiential Skill Modeling and Client Perspective

Coaches utilized AIC to refine their own coaching practice through observational and experiential learning, using the platform as a modeler of coaching techniques. Through interaction with the AI coach, coaches observed core competencies, such as questioning, paraphrasing, and metaphor application. Experiencing and observing AIC from a coachee perspective also encouraged a shift toward a more client-centered, facilitative style. Coach L highlighted the value of observing the AI coach: *‘I also learn how AI is coaching. How does it ask questions, how does it provide feedback, acknowledging what you said. Yeah, this is a very good experience and reference as well’*. Additionally, by assuming a coachee role, coaches gained firsthand insight into the client experience, a change in perspective they deemed critical, especially for novice coaches. Coach J emphasized this developmental benefit: *‘I thought that AI coaching could be a good training option for beginning coach learners… it’s very important to have client experience to be a coach. So, I think the AI coach will support us very well in that part.’*

#### 4.8.2. Supervision and Feedback, for Continuous Growth

Participants also leveraged AIC in roles similar to those of a coaching supervisor or mentor, identifying the technology as a vital resource for their ongoing professional growth and skill refinement. Coaches engaged with the platform as a neutral sounding board to process challenges encountered in their own human-led coaching sessions. Coach C described this supervisory function: *‘I would sometimes use it like my mentor coach. Sometimes I would struggle with my own clients’ sessions. So, I would type in keywords or maybe themes… and I would see what the AI would respond to that.’*

Additionally, coaches used the AI as a dynamic feedback provider, addressing the AI as a coachee that would offer them targeted evaluations of their skills as a coach. Coach K explained how this evaluative partnership provides a safe environment for practicing, assessing, and refining their coaching skills and facilitates professional development as a coach: *‘I hope that it can help me improve my coaching skills… Giving me feedback based on core competencies or some other coaching skills… I coach AI, and the AI gives me feedback.’*

### 4.9. AIC’s Role in Catalyzing Coaching Market Evolution: Demand Dynamics and the Premium of Human Expertise

The innovations in coaching delivery that AIC entails, including blended coaching, differentiated functions, and target groups, affect not only the professional identities of coaches but also transform the coaching market as a whole. Besides altering the mode of delivery of coaching to clients, AIC also bears the potential to influence the demand for coaching services in the market. Coaches indicated that the widespread adoption of AIC is fundamentally reshaping market perceptions. Rather than perceiving AIC as a threat, participants primarily framed it as a tool that increases the visibility and accessibility of coaching and bears potential for market growth. They anticipated that as individuals experience the benefits of AIC, demand for human coaching would likely increase, particularly as clients seek the unique value that human coaches provide, such as empathy and relational depth. Coach F reflected on this door-opening effect of AIC: *‘I also see a new opportunity for us human coaches to be of value, especially if AI coaching spreads more […]. If AI coaches spread more and people know how to use [a] coach better, then they can maybe begin to feel that this experience might be better with a human.’* Some participants, however, also expressed concern that potentially negative experiences with AIC could adversely affect the perceived quality of coaching overall. Coach A shared this concern: *‘If the quality of AI coaching is not very good, then people who experience coaching for the first time may be disappointed at coaching and then they would not use human coaching either.’* Nonetheless, the prevailing perspective emphasized AIC’s potential for market expansion. In this evolving landscape, AIC increases the availability of coaching through its instrumental accessibility, potentially paving the way for human coaching as a premium offering for complex, relational transformation.

Collectively, these findings reveal a sophisticated process of professional sensemaking, as coaches move from initial technological apprehension to a proactive reconstruction of their own occupational roles and the roles they ascribe to AIC. By reframing AIC as a collaborative supplement in blended models or alternative service provision with a differing scope (4.7), utilizing it as a developmental partner for professional growth (4.8), and anticipating its potential for market expansion (4.9), participants navigated the automation-augmentation paradox. This shift established a clear vocational hierarchy from the coaches’ perspective: AIC handles instrumental, structured tasks and expands market access, while human expertise remains the indispensable premium for complex relational transformations and nuanced psychological shifts. This transition from a skeptical to a strategic posture provides the empirical foundation for a deeper examination of how technology catalyzes identity work within the coaching profession.

## 5. Discussion

Our findings reveal that organizational adoption of AIC is not merely a technological shift; it involves a profound identity-driven negotiation process. The organizational context in which the top-down yet participatory implementation of AIC initially triggered significant tensions, pressures, and role ambiguity. These challenges manifested in a dynamic spectrum of ambivalent cognitive and emotional reactions among coaches. These initial reactions ranged from positive affective responses and appraisals of AIC as a strategic professional development opportunity and a means of career future-proofing, to negative responses of skepticism and technological uncertainty, as well as perceived threat to professional identity. Specifically, coaches expressed concerns about the erosion of their unique relational value, which is central to their human-centered vocation, and anticipated the risk of being replaced by AIC. As coaches transitioned from passive observers to active users, direct engagement with AIC enabled them to identify both key similarities between human and AI coaching in core coaching techniques, alongside fundamental differences in relational depth. However, coaches also recognized AIC’s distinct advantage in providing an easily accessible, psychologically safe space. This experiential realization allowed coaches to engage in active boundary work and to clarify roles, thereby resolving role ambiguity by delineating functional distinctions between human and AI coaching. This also implied more fundamental identity negotiations, resorting to both protective and expansive identity customization strategies. Ultimately, these negotiations resulted in the identification of three primary roles for AIC: delivering coaching to clients via blended and service-division models, serving as a development partner for coaches’ professional growth (e.g., skill modeling, feedback, and supervision), and driving the overall coaching market evolution by enhancing service visibility and shaping demand dynamics.

**Figure 2:**
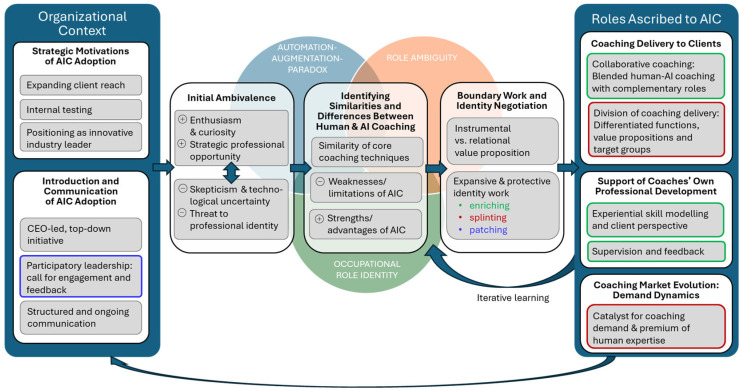
Human coaches’ adaptation to AI coaching: Conceptual framework.

To synthesize these dynamics, we present an integrative conceptual framework (Figure 2) that illustrates the multidimensional and processual nature of coaches’ vocational adaptation. The vocational adaptation progresses from *organizational triggers* through a central *negotiation zone*, where they grapple with the automation-augmentation paradox, attempt to reduce role ambiguity, and engage in protective and expansive identity work, to tangible outcomes manifested in *a set of clearly defined roles to AIC*. Finally, *two feedback loops* illustrate the dynamic nature of this process. One loop shows how outcomes inform and reshape the organizational context, while the other, internal loop, shows how these outcomes inform future adaptation, representing an iterative professional learning process.

***From a fear of automation to exploring the possibilities of augmentation.*** Coaches’ adaptation process reflected the nonlinear, paradoxical nature of professionals’ response to AI. While many coaches initially viewed AIC as a disruptive threat to their professional status and job security, especially as they witnessed AI replicating core coaching techniques, ongoing organizational support and opportunities for direct engagement gradually shifted their perspective. Coaches moved from a simple fear of replacement to a nuanced understanding of augmentation, seeing AIC as a resource for automating routine tasks and enabling greater focus on complex, relational client work, also through leveraging AIC for their own professional development. This evolving viewpoint, moving through cycles of apprehension and exploration, manifests how professionals must actively renegotiate their value in a changing technological landscape when confronted with the automation-augmentation paradox (Raisch & Krakowski, 2021).

***Protective and expansive identity work: Coaches utilized a full spectrum of identity customization strategies.*** The primary protective strategy was *splinting* (Pratt et al., 2006), whereby coaches anchored themselves to the prior stable identity of a traditional human coaching intervention. This reaction was evident when coaches emphasized their unique ‘humanness’ as irreplaceable. Specifically, coaches consistently argued for the irreplaceability of human qualities such as relational attunement, handling nuances, and seeing the broader context, exemplified by Coach L noting that, while AIC is good at coaching the ‘how,’ it cannot coach ‘the who, the person I am.’ However, recent meta-analytic evidence (Ong et al., 2025) complicates this perspective: Across several multi-study evaluations, third-party observers rated AI-generated support messages as more empathic and compassionate than those produced by human professionals. Yet, when raters learned that a message came from AI, their empathy ratings dropped significantly—a phenomenon labeled the ‘AI penalty’—while human-written messages gained an empathy advantage after source disclosure. These findings suggest that coach beliefs in the irreplaceability of human empathy may be shaped not only by actual qualitative differences but also by recipient expectations and attribution effects. That said, our data indicate that in real coaching settings, clients and coaches still place central value on authenticity, relational trust, and nuanced understanding—qualities currently more readily associated with human professionals. Thus, even as generative AI can replicate elements of empathic communication as measured in controlled single-time exchange studies, the social context and perceived source remain central to the perceived value of empathy in coaching.

Coaches did not simply adopt a defensive posture though. They also engaged in expansive identity work. Their strategies included *enriching* their skillset by reframing AIC as a professional development tool, for example, by using AI as a ‘technique modeler,’ to learn and improve coaching techniques; as an ‘enabler of client experience,’ to better understand the coachee’s perspective; and as a ‘coach supervisor or mentor,’ to reflect upon their coaching approach or, as Coach C shared, ‘to process client challenges.’ Furthermore, coaches engaged in *patching* by combining their role as coaching experts with a consulting role to improve the AIC service. Coach I exemplified this by emphasizing how she tested the AI, ‘providing feedback’ and ‘sharing back’ her observations to her organization. This broad spectrum of identity work demonstrates a sophisticated adaptation, moving from identity threat to a reconstructed, AI-augmented professional identity (Selenko et al., 2022; Sergeeva, 2023; Strich et al., 2021).

***From identity work to blended and service division models of coaching delivery: reducing role ambiguity through practice redesign.*** The tangible outcomes of coaches’ identity work were the creation of new blended coaching models that served to reduce the initial role ambiguity. The *protective* identity work, with coaches anchoring their value in their unique ‘humanness,’ directly led to a ‘service division’ model. By delineating their core, irreplaceable value, coaches could then design a work model in which distinct tasks are assigned to humans and AI based on complexity. As explained by Coach J, who envisioned human coaches handling ‘sensitive, executive kind of coaching,’ while AIC would manage coaching on ‘a certain subject or topic.’ Similarly, the *expansive* identity work of reframing AI as a tool for augmentation led to a ‘service enrichment’ model. Having enriched their own practice, coaches could then envision a blended approach in which they remained central to the client relationship, while ‘in the meantime, the AI coach checks the progress,’ as described by Coach A. The emergence of these distinct models of coaching delivery demonstrates a pragmatic resolution of the initial identity threat, showcasing a shift from a zero-sum conflict to a collaborative, human-led integration of technology (cf. Faulconbridge et al., 2024; Strich et al., 2021).

### 5.1. Theoretical Contribution

Our study contributes a vital, experience-based perspective on AI-driven change in workplace coaching by investigating the identity work through which professionals navigate this phenomenon. The theoretical contributions of our study are threefold. First, our study extends the automation-augmentation paradox. While prior work establishes the paradox as a central tension in the new world of work (e.g., Raisch & Krakowski, 2021), our findings reveal that its resolution in a relational field such as workplace coaching is not merely a task-allocation problem but an identity-driven process. This process involves ongoing negotiation, contextual boundary setting, and a dynamic redefinition of professional value propositions (Jarrahi, 2018; Schad et al., 2016). Coaches in our study navigated the paradox through protective and expansive identity work, simultaneously fearing automation while embracing augmentation. We demonstrate that, for knowledge-intensive work like coaching, the automation-augmentation paradox is experienced and resolved at the level of professional identity.

Second, we contribute to occupational role identity theory by providing a fine-grained, empirical account of the specific identity customization strategies professional coaches use to adapt to AIC (cf. Pratt et al., 2006). By documenting these mechanisms within the blurry field of coaching, our study addresses a gap in the literature, which has primarily focused on highly regulated fields. We demonstrate that in a more fluid profession, identity customization can be particularly dynamic. For example, coaches engaged in *enriching* their practice, by using AIC as a ‘technique modeler’ to observe questioning styles, or an ‘enabler of client experience’ to better understand the coachee’s perspective. Furthermore, they demonstrated *patching* by combining their role as coaching experts with a consulting role to improve the AIC service. We thus extend the theory by demonstrating how these strategies are used not just to defend an existing identity but to proactively construct a new, enduring AI-augmented professional identity.

Third, our study refines theories of role ambiguity. While much of the literature frames role ambiguity as a negative stressor to be eliminated (e.g., Kahn et al., 1964; Rizzo et al., 1970), our findings demonstrate a more generative outcome, highlighting role ambiguity as a potential catalyst for vocational adaptation. Vocational adaptation to AI is a dynamic and negotiated process. In the organizational context, organizational scaffolding appears to be key in fostering a shift from defensive postures toward proactive exploration. Ambiguity became a necessary space for coaches to engage in proactive boundary work, leading to boundary setting, role clarification, and the development of different novel blended coaching models of coaching delivery, as well as new ways of supporting continuous professional growth. We thus show that managed ambiguity can be a feature, not a bug, in professional adaptation, a finding that builds on recent evidence of ambiguity’s positive potential (Martínez-Díaz et al., 2020) and the iterative nature of adaptation, which aligns with contemporary boundary work frameworks (Martínez-Díaz et al., 2020; Strich et al., 2021).

### 5.2. Practical Implications

Our findings offer practical implications for coaching organizations and individual coaches as they navigate their adaptation to and integration of AIC. For coaching organizations and their leaders, our study highlights that successful AIC adoption is a process of managed organizational change, where role ambiguity needs to be contained, rather than simply a technical implementation. To mitigate initial identity concerns and address the role ambiguity inherent in this transition, leaders should position AI as an augmentative tool rather than a replacement. By explicitly communicating how AIC serves to enhance and complement human expertise, organizations can directly confront the core tension of the automation-augmentation paradox. Furthermore, leaders should establish ‘organizational scaffolding’ for effective sensemaking. Instead of imposing an exclusively top-down mandate, this approach involves fostering spaces for collective adaptation, which includes facilitating ongoing dialogue and establishing working groups that provide real-time feedback and explore best practices for new service delivery and professional development models. Finally, organizations should prioritize training focused on adaptive professional competencies. This approach entails moving beyond merely technical skills to empower coaches to reflect, articulate, and leverage their uniquely human contributions, and develop the strategic capabilities necessary to design and implement effective blended coaching models.

These implications also extend to coach training. In addition to imparting knowledge and technical skills related to AI tools for coaching, coach trainers should facilitate opportunities for coaches to engage in identity work. Coach trainers are encouraged to prompt reflection on identity customization strategies and to challenge coaches to develop a nuanced perspective on AI in coaching, fostering both critical self-awareness of coaches’ own defensive responses and caution against uncritical enthusiasm for AIC.

For professional coaches, our findings emphasize that they are not passive recipients of technology; instead, they hold significant agency in shaping their professional trajectory. Coaches should engage in proactive identity work, which involves both a protective aspect, by identifying and strengthening core human skills that AI cannot replicate, and an expansive element, by actively seeking growth opportunities. A key strategy is to experiment with AIC as a tool for professional development, reframing AIC from a rival to a resource by using it as a ‘technique modeler,’ an ‘enabler of client experience,’ or as a confidential ‘coaching supervisor or mentor.’ Furthermore, coaches can become the architects of blended coaching models by taking the lead in designing new service offerings. Indeed, Terblanche et al. (2024) showed that coach attitudes play a pivotal role in the effectiveness of AIC in augmenting human coaching in blended coaching delivery models. By identifying where AI can handle routine tasks and where human intervention is essential, coaches can redefine their value proposition and create more effective and scalable coaching solutions.

### 5.3. Limitations and Future Research

Despite its contributions, this study has several limitations that suggest avenues for further investigation into the adoption of AIC in professional practice.

First, our study was intentionally designed as an in-depth inquiry within a single Asian coaching company. While this focus offers a needed in-depth, nuanced perspective on AI adoption in the workplace, it also defines the study’s boundaries. Our sample was designed to prioritize analytic depth over breadth, allowing us to reach meaning saturation and develop a conceptual model. Accordingly, future research is warranted to test the model’s applicability across diverse contexts, for example, with broader coach populations across diverse geographic regions and organizations. Second, this study primarily utilized single-point interviews with coaches. Although we intentionally included retrospective questions to explore participants’ evolving experiences with AIC, the study cannot fully encompass all the processual, longitudinal dynamics of adaptation. Coaches recounted their initial impressions and reactions when they first learned about AIC adoption during the kick-off and their perceptions after several months of interaction. Additionally, pre- and post-adoption interviews with key organizational stakeholders, including the AIC provider and the company’s CEO, captured changes at critical organizational levels. However, processes (e.g., automation-augmentation cycles, or identity negotiations) may evolve over extended periods, requiring further research. Therefore, future research should employ longitudinal designs to track coaches throughout the AIC implementation and adaptation trajectory, allowing for a more nuanced understanding of how professional roles, boundaries, and organizational responses evolve over longer time frames.

Third, our focus on the perspectives of professional human coaches, while insightful for exploring identity work and adaptation strategies, necessarily pulls focus from the broader AIC ecosystem. Key stakeholders such as coach training providers, accrediting bodies, professional associations, and client organizations play critical roles in shaping the adoption, institutionalization, and normalization of AI in coaching—as do coachees (Terblanche et al., 2024) and client organizations on the demand side of the coaching market. Integrating these perspectives would yield a more holistic view of the negotiation and evolution of standards, competencies, and values as AIC continues to shape the coaching profession.

## 6. Conclusions

The adoption of AIC in organizational settings acts as both a catalyst for professional disruption and a potent driver of renewal. Our study demonstrates that human coaches respond to the challenges and opportunities of AIC by actively reworking their occupational identities. Through a dual process of protective and expansive identity work, they creatively manage the evolving boundaries between human and machine expertise, culminating in the development of new models of coaching delivery and professional growth.

This negotiation of core human-centric elements, such as empathy and contextual understanding, occurs at the individual and collective level within the organization, but also as part of a collective adaptation within the profession at large. By providing an in-depth account from an Asian context, our research also contributes a vital non-Western perspective to this global conversation. Adapting to AIC is thus a dynamic and social process, fundamental to the ongoing evolution of coaching in the age of intelligent technologies. Ultimately, this research advances understanding of how AIC is reshaping the essence of professional human coaching, foregrounding the ongoing negotiation of identity, value, and expertise in an increasingly technology-driven world.

## Figures and Tables

**Table 1:** Interviewees’ demographics, coaching experience, and AI coaching (AIC) engagement.

ID	Age	Sex	Role	Tenure (Years)	Education	Coaching Exp. (Years)	No. of AIC Sessions	Cultural Background
A	38	F	Coach; R&D Leader	6	PhD, Social Psychology	*NA*	4	Japanese
B	65	M	CEO Coaching Company; Exec. Coach	25	MA, Clinical Psychology	25	12	Japanese
C	23	F	Coach; Sales	1	BA, Business	1	8	Mixed
D	37	M	Coach; Sales;	1	BA, Commerce	1	3	Japanese
E	40	M	Coach; Sales	1	MBA	1	1	Japanese
F	31	M	Coach; Sales	8	BA, Economics	8	14	Mixed
G	28	F	Coach; Marketing	5	BA, Communication	5	15	Mixed
H	38	F	Coach; R&D Mentor	9	MA, Human Resources Management	8	4	Chinese (HK)
I	39	F	Exec. Coach; Team leader	10	MA, Education	8	15	Thai
J	66	F	Exec. Coach	26	BA, English Literature	26	8	Japanese
K	56	F	Coach; Recruitment	24	MA, Linguistics	5	4	Japanese
L	46	F	Exec. Coach; Manager	9	MBA	9	5	Chinese
M	29	M	CEO AIC Provider; Project Manager	3	MA, Economics	NA	NA	Ukrainian

*Note:* AIC sessions lasted 30 min each. Mean age = 42; mean AIC sessions = 7.6, Exp. = experience.

**Table 2:** Summary of categories and subcategories for the organizational context of AIC adoption shaping coaches’ experience.

Subcategory	Brief Description
*4.1. Strategic Motivations of the Organization to Adopt AIC*	
4.1.1. Expanding Client Reach	Leverage AIC for scalability across geographical regions and target groups to reach segments underserved by human coaching. Enable wider market uptake by reducing domestic cultural barriers associated with human coaching.
4.1.2. Internal Testing	Allow user experience and familiarization to build staff proficiency and comfort with AIC before client deployment. Pilot internally to assess quality and improve iteratively through continuous user feedback.
4.1.3. Positioning as an Innovative Industry Leader	Reinforce industry leadership and market differentiation by pioneering technological innovation.
*4.2*. *Organizational Processes for the Introduction and Communication of AIC Adoption*	
4.2.1. CEO-Led, Top-Down Initiative	Top-level sponsorship driving engagement by framing AIC as a strategic initiative.
4.2.2. Participatory Leadership: Call for Engagement and Feedback	Invite staff to test AIC and provide feedback for development.
4.2.3. Structured and Ongoing Communication	Utilize company-wide announcements and frequent progress updates to clarify roles, expectations, and maintain implementation momentum.
*4.3*. *Coaches’ Initial Emotional and Cognitive Reactions*	
4.3.1. Enthusiasm and Curiosity	Positive affective responses; openness to try new technology.
4.3.2. Strategic Professional Opportunity	Cognitive appraisal of AIC as a vehicle for vocational innovation and career future-proofing.
4.3.3. Skepticism and Technological Uncertainty	Lack of familiarity with AIC leading to cautious expectations and difficulty imagining practical application.
4.3.4. Perceived Threat to Professional Identity	Anxiety over role displacement and erosion of human relational value.

**Table 3:** Key comparative dimensions of human and AI coaching.

Category	Brief Description
*4.4. Similarities Between AIC and Human Coaching*	
Shared Core Coaching Techniques	Human and AI coaching utilize the same core coaching techniques, such as questioning, summarizing, and acknowledgment, to facilitate a non-directive, reflective process.
*4.5. Weaknesses and Limitations of AIC*	
4.5.1. Lack of Holistic Exploration and Adaptive/Flexible Conversation Flow	AIC’s structured, goal-focused approach restricts conversational flexibility, struggles to navigate ambiguity and coachees’ evolving topics, and fails to address the coachee as a whole person.
4.5.2. Lack of Emotional Resonance and Relational Depth	AIC lacks the capacity to grasp implicit emotional cues or create a genuine sense of being ‘heard,’ leading to reduced social resonance that can hinder coachee commitment.
4.5.3 Difficulty Supporting Novice Coachees Lacking Coaching Literacy	AIC’s inability to adapt its approach to a coachee’s ‘coaching literacy’ makes it less helpful for beginners.
*4.6. Strengths and Advantages of AIC*	
4.6.1. Psychological Safety: Freedom of Expression and Neutrality	AIC’s perceived neutrality and absence of own agenda/feelings, combined with the user’s control over the pace, creates a non-judgmental space that reduces social pressure and allows more honest, shame-free reflection.
4.6.2. Accessibility and Instrumental Utility	AIC’s on-demand availability, scalability, and informational resourcefulness enable affordable, accessible, and targeted coaching that provides immediate, any-time support to substantially enlarged target groups.

**Table 4:** Roles human coaches ascribe to AIC.

Category	Brief Description
*4.7*. *AIC’s Role in Delivering Coaching to Clients*	
4.7.1. Collaborative Coaching Delivery: Blended Human–AI-Coaching	Embracing AIC as a collaborative supplement, establishing a blended model of joint coaching delivery where AI automates routine, goal-focused tasks to augment the impact of human coaching.
4.7.2. Division of Coaching Delivery: Differentiating Functions, Value Propositions, and Target Groups of Human vs. AI Coaching	Independent coaching delivery where humans and AI operate independently, each according to their distinct value propositions. Human coaches address complex contexts, provide relational depth, and serve executives, whereas AIC offers scalable support for structured topics.
*4.8*. *AIC’s Role for Coaches’ Own Professional Development*	
4.8.1. Experiential Skill Modeling and Client Perspective	Using AIC as a modeler of core coaching techniques (e.g., questioning and metaphors) and leveraging the coachee role to gain firsthand insight into the client experience.
4.8.2. Supervision and Feedback, for Continuous Growth	Engaging with AIC as a mentor or supervisor to process client-related challenges and receive targeted, competency-based feedback.
*4.9*. *AIC’s Role in Catalyzing Coaching Market Evolution*	
Demand Dynamics and the Premium of Human Expertise	AIC serves as a catalyst for market growth by increasing the visibility and accessibility of coaching services. Despite potential risks to the profession’s perceived quality, AIC is perceived as a “door-opener” repositioning human expertise as a premium service in an evolving market.

## Data Availability

The data used in this study are confidential and are not accessible to the public. However, the authors can provide the data upon reasonable request, subject to certain restrictions.

## Abbreviations

The following abbreviations are used in this manuscript:

AI	Artificial Intelligence
AIC	Artificial Intelligence coaching
RQ	Research Question
QCA	Qualitative Content Analysis
